# Do Menstrual Hygiene Management Interventions Improve Education and Psychosocial Outcomes for Women and Girls in Low and Middle Income Countries? A Systematic Review

**DOI:** 10.1371/journal.pone.0146985

**Published:** 2016-02-10

**Authors:** Julie Hennegan, Paul Montgomery

**Affiliations:** Centre for Evidence-Based Intervention, University of Oxford, Oxford, United Kingdom; University of Exeter, UNITED KINGDOM

## Abstract

**Background:**

Unhygienic and ineffective menstrual hygiene management has been documented across low resource contexts and linked to negative consequences for women and girls.

**Objectives:**

To summarise and critically appraise evidence for the effectiveness of menstruation management interventions in improving women and girls’ education, work and psychosocial wellbeing in low and middle income countries.

**Methods:**

Structured systematic searches were conducted in peer-reviewed and grey literature to identify studies evaluating education and resource provision interventions for menstruation management. Individual and cluster randomised controlled trials were eligible for inclusion, as were non-randomised controlled trials. Study characteristics, outcomes and risk of bias were extracted using a piloted form. Risk of bias was independently assessed by two researchers.

**Results:**

Eight studies described in ten citations were eligible for inclusion. Studies were highly heterogeneous in design and context. Six included assessment of education-only interventions, and three provided assessment of the provision of different types of sanitary products (menstrual cups, disposable sanitary pads, and reusable sanitary pads). A moderate but non-significant standardised mean difference was found for the two studies assessing the impact of sanitary pad provision on school attendance: 0.49 (95%CI -0.13, 1.11). Included studies were heterogeneous with considerable risk of bias. Trials of education interventions reported positive impacts on menstrual knowledge and practices, however, many studies failed to assess other relevant outcomes. No trials assessed or reported harms.

**Conclusions:**

There is insufficient evidence to establish the effectiveness of menstruation management interventions, although current results are promising. Eight trials have been conducted, but a high risk of bias was found and clinical heterogeneity precluded synthesis of most results. Whilst trials provided some indication of positive results, further research is needed to establish the role of menstruation hygiene management in education performance, employment and other psychosocial outcomes. This review provides a concise summary of present trials and highlights improvements for future work.

## Introduction

The effective, hygienic management of menstruation is essential for women and girls to participate in society with dignity and comfort. Effective menstrual hygiene management (MHM) includes access to clean absorbents, with facilities to change, clean or dispose of these as needed, and access to soap and water for cleaning the body and absorbents.[1] Studies across low and middle income countries (LMICs) have reported that more than 50% of girls have inadequate MHM, with higher proportions reported in rural areas.[2–5]

Health and social research has only recently sought to address the neglect of MHM as a significant development issue and barrier to achieving gender equality. Both qualitative and quantitative work has suggested that poor MHM results in school absenteeism, distraction, and disengagement.[6,7] There is a shortage of literature addressing the impact of MHM on adult women, but poor MHM is also likely a barrier to occupational attendance and engagement.[8,9] Focus groups from a factory in Pakistan suggested that women missed up to three days’ work per month due to menstruation.[9]

Other programs of qualitative work have reported psychosocial consequences of poor MHM (e.g.,[10]). Feelings of shame, fear (and fear of stigma), anxiety, and distraction have been described across a range of contexts.[11,12] Few studies have quantified the relationship between MHM and psychosocial consequences. Studies that have been conducted have often focused on menarche, reporting that high proportions of girls feel unprepared and afraid at this time.[13–15]

### MHM Interventions

Interventions to address MHM have been categorised into two groups; *hardware interventions* designed to address material deprivations such as the provision of absorbents, or improved Water, Sanitation and Hygiene (WASH) facilities; and s*oftware interventions* which address deficits in knowledge of menstruation and management by providing education.

#### Hardware interventions

The cost and availability of sanitary products, and underwear in which to wear them, is a fundamental barrier to MHM.[16–18] Commercial absorbents are frequently unavailable or too expensive.[2,16,17,19,20] The provision of clean sanitary products (e.g., commercial or home-made pads) addresses this material deprivation and is hypothesised to reduce discomfort, and concerns regarding soiling outer garments. Improved management and comfort may also reduce associated stigma, ridicule, and embarrassment which deters women and girls from attending school or work. In Ghana, Dolan and colleagues [21] found that over three quarters of schoolgirls surveyed reported soiling outer garments during their last menses, and found that school attendance improved by 9% after 5 months with the prevision of disposable sanitary pads.[18]

Inadequate WASH represents a barrier to MHM, particularly the ability to clean absorbents, and the body, in private.[22] The design of toilets often fails to meet women’s physical and psychological needs.[21,23] Latrines lacking doors or locks may threaten safety and cause embarrassment.[6,24] Hygiene guidelines recommend changing absorbents every two to six hours dependent on blood flow, thus facilities are needed both at home and in schools or workplaces.[17] Improvements to WASH access may enable women to clean reusable absorbents and genitals hygienically, as well as reduce discomfort and embarrassment. Open pit toilets or toilets without disposal facilities mean blood or used sanitary products reveal when a woman is menstruating, resulting in embarrassment and stigma.[23,25] ‘Girl friendly’ changes such as gender-separate latrines, locks on toilets, discrete facilities for changing absorbents or washing have also been hypothesised to enable improved MHM.

#### Software interventions

Adolescent girls have been found across a range of countries to lack knowledge of the physiology and management of menstrual bleeding.[5,13,26] Ali and Rizvi [13] reported that in Pakistan, less than 50% of girls surveyed received information about menstruation prior to menarche, only 15% information about management. Cultural beliefs and taboos may also contribute to poor MHM through the perpetuation of misinformation or unhygienic customs. Studies across contexts have reported taboos around the disposal of menstrual blood, and practices including restrictions to bathing and participation in social activities.[12,14–16,27]

Improving understanding of menstruation, such as knowledge of cycle length and requirements for hygiene, is hypothesised to improve management practices, self-efficacy, and reduce anxiety.[17,28–30] In this way education interventions may facilitate school attendance and engagement in the classroom through improved ability to effectively manage menstruation, and confidence in management methods. This education may also reduce negative psychosocial consequences by normalising menses and dispelling myths.[18,21]

### Previous reviews

Sumpter and Torondel [31] conducted the only systematic review of MHM interventions, focusing on health outcomes. The review included some education and social outcomes, but had a number of limitations. First, pre-post comparisons and observational studies were synthesised alongside randomised controlled trials despite representing different risks of bias. Second, the review searched only peer-reviewed journals thus missed the wider ‘grey’ literature. Third, only limited terms ‘school’ and ‘educat*’ were used to search for education outcomes. Only ‘social restrictions’ such as restricting diet or interactions during menstruation, rather than psychosocial consequences described in the literature, were included. The review did not discuss the inclusion or exclusion of WASH interventions. Finally, the review search was conducted in May 2012, with at least two trials published since this time.[18,32,33]

Two other relevant reviews have been conducted. Birdthistle and colleagues [34] and Jasper and colleagues [35] focused on WASH interventions. Neither identified all relevant studies, nor appraised the risk of bias in included studies.

### The present review

Hardware and software interventions have been employed by governments, international organisations and local charities seeking to address poor MHM and associated consequences.[20,36] These programs have been enacted in the absence of evidence for their effectiveness. Given the weak evidence base and ongoing plans to implement MHM interventions, the present work will provide an essential appraisal of eligible studies and highlight implications for future research.

This review will summarise and critically appraise evidence for the effectiveness of hardware and software interventions for MHM aimed at improving education, employment or psychosocial outcomes for females in LMICs.

## Methods

This review was conducted according to the requirements of the Preferred Reporting Items for Systematic Reviews and Meta-Analyses (PRISMA) statement.[37] Review protocol was registered on PROSPERO (CRD42014010631) http://www.crd.york.ac.uk/PROSPERO/display_record.asp?ID=CRD42014010631.

### Criteria for selecting studies

Individually and cluster randomised controlled trials (RCTs/cRCTs) were eligible for inclusion, representing the most rigorous evidence with the ability to infer causality.[38] Non-randomised controlled trials and controlled before-after studies were also included despite greater potential for bias,[38] due to the lack of available trials and difficulty conducting RCTs in LMICs.[39] Cross-sectional studies and pre-post designs which did not include a control group were ineligible.

Studies were eligible if they reported on outcomes for menstruating females from LMICs as defined by the World Bank [40] (http://data.worldbank.org/about/country-classifications).

### Eligible interventions

#### Hardware interventions

1. The provision of clean absorbents/sanitary products (disposable or reusable),
2. Improved WASH or girl-friendly facilities. Key aspects of WASH interventions relevant to menstruation were derived from the WHO [41] report on *Water*, *Sanitation and Hygiene standards for schools in low-cost settings*, the *UN Gender*, *Water and Sanitation Policy Brief*.[42] Studies could include any number of these aspects where authors’ hypothesised that the intervention was sufficient to improve MHM practices:
  - Improved clean water supply for menstruation management (e.g., access to water within latrine or private areas);
  - Provision of soap or disinfectant for body and absorbent cleaning;
  - Improved absorbent disposal facilities;
  - Improvements to latrine privacy or safety.

#### Software interventions

Interventions that delivered sufficient education to provide an understanding of the biological process of menstruation (e.g., the cyclic nature of menses, origin of menstrual blood), and which authors hypothesised was sufficient to improve either MHM practices or misconceptions and confusion. Information could be provided in person or via printed or electronic resources.

### Eligible outcomes

#### Primary outcomes

Education. School attendance (full or partial days) from school records or self-reported.
Employment. Self- or employer-reported attendance/absenteeism (full or partial days).
Psychosocial Outcomes. Outcomes were derived from qualitative literature identifying psychosocial consequences of poor MHM and included; anxiety, confusion, depression, embarrassment, emotional distress, fear, powerlessness, self-confidence, self-efficacy, shame, stigma, worry, and self-imposed withdrawal from activities such as playing sport. Externally enforced restrictions (e.g., not attending religious ceremonies) were not eligible outcomes as interventions were not hypothesised to impact such traditions.

#### Secondary outcomes

Education. Academic achievement and school engagement.
Employment. Measures of productivity, engagement, and worker satisfaction.
Psychosocial outcomes. Other measures of attitudes related to menstruation.
Menstrual knowledge and management. Knowledge of menstruation and improved management practices.

### Search methods

Searching strategy was based on inclusion criteria and developed with reference to searches reported in previous reviews.[11,31,34] Searches were conducted in English, but no restrictions were set for language, date, or publication type. Searching strategy for PsycINFO is displayed in Table 1.

**Table 1:** PsycINFO search strategy.

**Search #1:** exp menstruation/ OR (menstrual period OR menstru* or menses OR catamenia OR menarche).mp. **Search #2:** hygiene/ OR water deprivation/ OR water safety/ OR water supply/ OR health education/ OR exp school facilities/ OR exp intervention/ OR (Management OR hygiene OR hygienic OR product* OR absorb* OR WASH OR sanitary OR sanitation OR toilet facilities OR bathroom facilities OR toilet* OR latrine* OR privy OR water closet OR lavatory* OR girl friendly OR wom#n friendly OR gender-separate OR privacy OR private OR dispos* OR waste OR intervention*).mp. **Search #3:** employee absenteeism/ OR school attendance/ OR exp school dropouts/ OR exp student engagement/ OR exp academic achievement/ OR exp occupational success/ OR exp productivity/ OR academic failure/ OR education/ OR academic self concept/ OR distraction/ OR colleges/ OR exp schools/ OR job performance/ OR employee efficiency/ OR employee productivity/ OR employee retention/ OR employee motivation/ OR employee engagement/ OR (absent* OR academic achievement OR academic performance OR attend* OR attention OR college* OR disengage* OR distract* OR educat* OR employee absenteeism OR employee engagement OR employee productivity OR engage* OR job OR productivity OR school* OR university OR vocation* OR work).mp. **Search #4:** agency/ OR anxiety/ OR depression, emotion/ OR distress/ OR embarrassment/ OR empowerment/ OR Health Knowledge/ OR Health Attitude/ OR shame/ OR fear/ OR mental health/ OR self determination/ OR exp interpersonal control/ OR exp self concept/ OR self confidence/ OR self efficacy/ OR self esteem/ OR self perception/ OR social isolation/ OR social acceptance/ OR exp stress/ OR well being/ OR (agency OR alone OR anxiety OR anxious OR attitude* OR confidence OR confused OR confusion OR depress* OR distress OR exclu* OR embarrass* OR empower* OR fear OR fearful OR isolation OR knowledge OR mood OR mental health OR psycholog* OR psychosocial OR restrict* OR secrecy OR shame OR social OR stress OR stigma OR self confidence OR self-efficacy OR self efficacy OR self-esteem OR self esteem OR upset OR understanding OR well-being OR well being OR worry OR worries).mp. **Search #5:** Search #3 OR Search #4 **Search #6:** Search #1 AND Search #2 AND Search #5

The following databases were searched from inception to present in January 2015:

- ASSIA (Applied Social Science Index and Abstracts)
- CENTRAL (Cochrane Central Register of Controlled Trials)
- CINAHL
- EMBASE
- Global Health
- IBSS (International Bibliography of the Social Sciences)
- MEDLINE
- OpenGrey
- POPLINE
- PsycINFO
- Science Citation Index
- Social Sciences Citation Index (SSCI)
- TRoPHI (Trials Register of Promoting Health Interventions)
- WHOLIS
- World Bank e-library

#### Searching other resources

Government departments and multilateral organisations were identified through the *Menstrual Hygiene Matters* report [17] co-publishing agencies, and websites and databases were searched. Handsearching was conducted in two key journals, reference lists of included studies, and the *Menstrual Hygiene Matters* report.[17] Subject experts were contacted to identify published, unpublished, or ongoing research.

### Data collection and analysis

Two reviewers independently screened titles, abstracts and full text articles. Included articles were determined by consensus. Data were extracted regarding participants, methods, intervention, analyses, outcomes, and risk of bias using a piloted form.

Risk of bias was assessed independently by both authors using the tool outlined in the *Cochrane Handbook*.[38] Additionally, three domains recommended by the Cochrane EPOC group [39] were used to assess threats to validity; imbalance of the outcome measures at baseline, comparability of intervention and control group characteristics at baseline, and protection against contamination. These were incorporated due to the inclusion of non-randomised studies and risk of contamination in software interventions.

Narrative synthesis is presented for included studies with standardised measures of effect calculated to aid comparison. For studies with multiple intervention groups, data were extracted for all comparisons. Where interventions were comparable (e.g., education by different providers), groups were combined to generate an average intervention effect.

Due to intervention and methodological heterogeniety data synthesis was possible for only one comparison, with two studies. Randomised and non-randomised studies were considered separately, as these designs represented different levels of risk of bias.[38] Review Manager 5.3 [43] was used to conduct meta-analysis using the generic inverse variance method to enable inflation of standard errors for clustering. Intra-cluster correlation coefficients (ICCs) were obtained from study reports or requested from authors. There were insufficient trials to conduct subgroup or sensitivity analyses.

## Results

**Fig 1:**
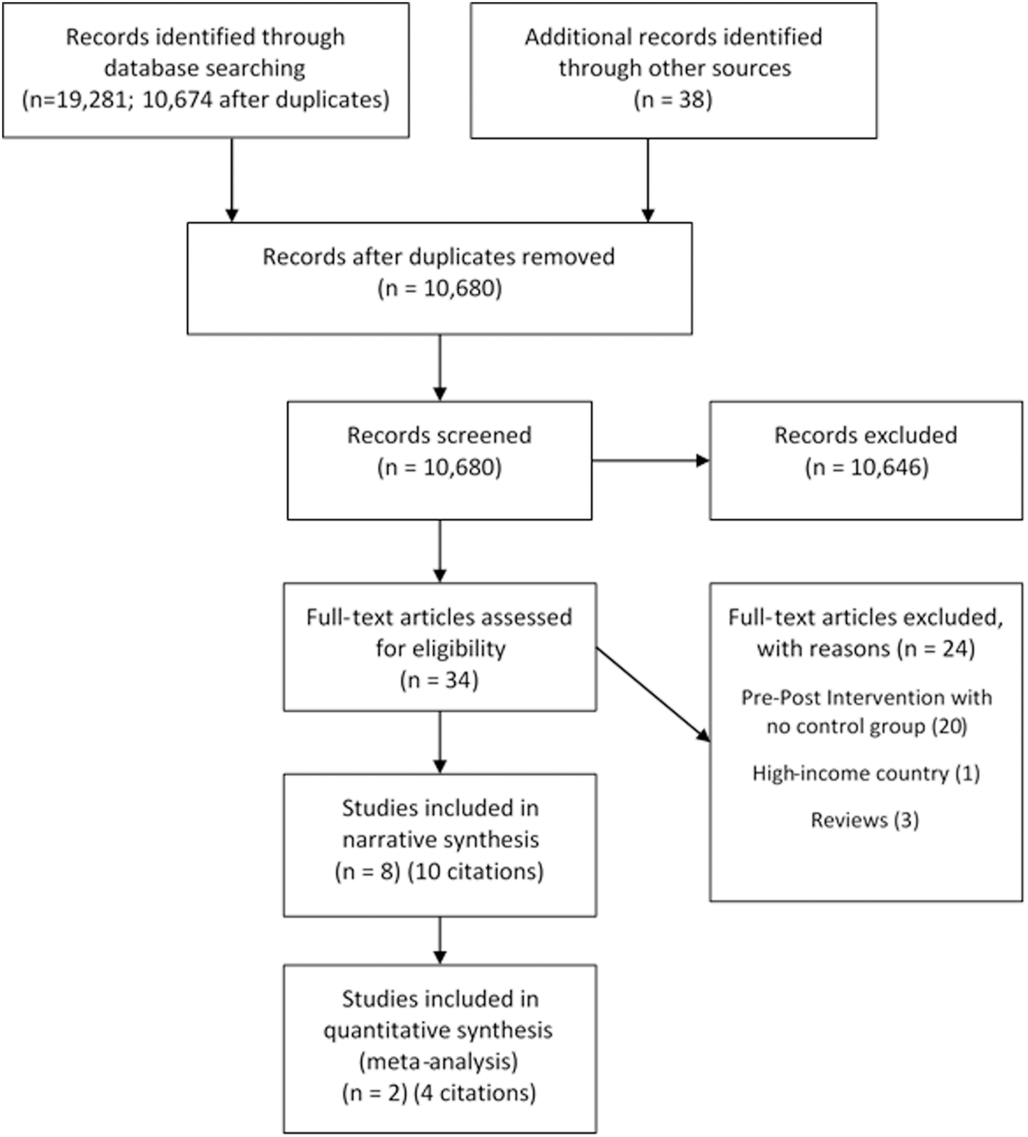
Study Flow Diagram [37].

After duplicates were removed a total of 10,674 titles and abstracts were screened (see Fig 1).

### Included studies

Eight trials were eligible for inclusion. Five evaluated education interventions, two the impact of providing sanitary products, and one included both interventions. One study assessed menstrual knowledge for both males and females. Authors were contacted, but unable to provide separated comparisons.[44] To provide an overview of available interventions, this study was retained although no outcomes could be reported.

Study characteristics are presented in Table 2. Six countries were represented; Iran (3), Saudi Arabia, Zimbabwe, Ghana, Nepal, and Kenya. Sample sizes ranged from 120 to 1823. Five studies included only girls post-menarche.[18,32,45–47] Exclusion and non-participation rates were typically not reported, although the majority of papers implied that few invited girls declined to participate. Studies typically included school-aged girls, (approximately 11–18 years). Wilson and colleagues reported an age range of 11–26, although the study was set in a secondary school with a mean participant age of 16 years.[32,33] One study focused on university students.[45] This study included only students who reported experiencing dysmenorrhea (three menstrual cramps in the past six months).

**Table 2:** Included study characteristics, interventions, and outcomes assessed.

Study	Study Design	Country/Setting	Sample	Intervention^1^	Duration	Description of Intervention
**Software Interventions**						
**Abedian 2011 [45]**	iRCT	Iran/Urban University	165 dysmenorrheic females aged 19–25 who had experienced menstrual cramps 3 or more time in the past 6 months.	Peer-led and health-provider-led ‘self-care’ education	Not reported	‘Self-care’ education session conducted via small-group discussion led by either a health-provider (midwife) or peer-leader. Sessions included the provision of information about menstruation and dysmenorrhea. ‘Self-care agency’ aspects of the session encouraged self-care behaviours including: searching for knowledge, expression of emotion, seeking assistance, control over external factors, self-control, and resource utilisation.
	**Timing of outcome assessment:** After 1 menstrual period. Final assessment after 2 menstrual periods (approx. 2 months)					
	**Outcomes assessed:** Self-report questionnaire: *Premenstrual dysmorphic syndrome symptoms* (DSM-IV-TR diagnostic criteria). *Menstrual Knowledge Questionnaire* (MKQ) developed by authors based on gynaecological understanding of menstruation. *Menstrual Attitude Questionnaire* (MAQ) [50] which included 5 sub-scales: menstruation as a debilitating event, menstruation as a bothersome event, menstruation as a natural event, anticipation and prediction of the onset of menstruation, and denial of any effect of menstruation. *Dysmenorrhea symptoms*: ratings of pain, menstrual blood loss, use of pain killers.					
**Djalalinia 2012 [48]**	iRCT	Iran/Urban middle schools	1823 females aged 11–15 (52.7% post-menarche by final evaluation).	Parent-led or school health trainer-led menstruation education	Not reported	Menstrual health education (unclear content, duration and format)
	**Timing of outcome assessment:** 2 years post-intervention					
	**Outcomes assessed:** Self-report questionnaire: *Menarche experience*: feelings regarding menarche including (Confusing, scared, uncomfortable, good feeling) as well as appropriate actions (e.g., being informed and prepared by female relative or doctor). *Menstrual information*: Information resource, attitude about adolescent health education requirements. *Menstrual and hygiene practices*: Proper menstrual hygiene (bathing and washing during period of menstruation, use of sanitary pad or cotton). *Menstrual pain*: Referral to doctor if experiencing pain					
**Fakhri 2012 [46]**	NRS. Controlled before after (clusters assigned)	Iran/Urban, rural high schools with low socio-economic status	698 post-menarche females aged 14–18	Youth and School Health Department-run puberty and menstrual health education	10x2hr education sessions	Menstrual health education provided by the Youth and School Health Department. Educational topics included the significance of adolescence, physical and emotional changes during adolescence, pubertal and menstruation health and premenstrual syndrome. Trainers employed an educational manual developed by an adolescent health professionals team.
	**Timing of outcome assessment:** Immediately post-intervention					
	**Outcomes assessed:** Self-report questionnaire: 71 item questionnaire in five sections: demographic characteristics, behaviours during menstruation, menstrual-related patterns, sources of information about menstruation and questions related to personal health. Reported outcomes include: *Menstrual health behaviours* including bathing, genital hygiene, *Menstrual attitude* including viewing menstruation as positive troubling or both). Primary outcome variable was *menstrual health* formed by collapsing across varied health behaviours and attitudes.					
**Fetohy 2007 [47]**	cRCT	Saudi Arabia/ Urban secondary school	248 post-menarche females aged 14–17	Education sessions aimed at increasing menstrual knowledge and healthy practices	1x120 minute session	Education session run by school nurse and two social workers aimed at increasing menstrual knowledge and knowledge of healthy practices and promote healthy practices and positive behaviour change: 1: General information and defining menstruation; 2: Causes of pain, abnormal menstruation and how to manage; 3:Normal changes and strategies for management of pain and menses; 4: Medical pain relieve information; 5: Types of food that should be consumed during menstruation
	**Timing of outcome assessment:** Immediately post intervention					
	**Outcomes assessed:** Self-report questionnaire: *Menstrual knowledge*: knowledge of definition, duration, age, pain, methods for relieving pain, hygiene. *Menstrual attitude*: attitude towards healthy and unhealthy practices. *Menstrual behaviour questionnaire*: management practices and hygiene eg. Bathing					
**Mbizvo 1997 [44]**	cRCT	Zimbabwe/Urban and rural high schools	1689 males and females (856 female) aged 10–19.	Puberty, menstrual and reproductive health education through leaflets, posters and pamphlets	Not reported	Information, education and communication materials in the form of leaflets, posters and pamphlets were produced to cover main areas of: Male reproductive function, sexuality, STDs and AIDS; female reproductive function, anatomy and STDs; human sexuality and responsible behaviour; unwanted/unplanned pregnancy and contraception; career posters, unspoilt with unplanned pregnancy
	**Timing of outcome assessment:** 5-months post-intervention.					
	**Outcomes assessed:** Self-report questionnaire. *Menstrual knowledge* correct menstrual practice and interpretation, menstruation as natural process not an illness, use of clean absorbent material. Reproductive and sexual health and behaviour					
**Hardware and Software Intervention**						
**Montgomery 2012 [18,21]**	NRS. Non-randomised cluster control trial	Ghana/Peri-urban and rural schools	120 post-menarche females aged 12–18.	Provision of disposable sanitary pads and puberty education, or education alone.	5 months	Sanitary pad provision: provision of underwear and 12 pads per month for the duration of the study. Received daily calendar to record menstrual cycle, as well as pencil and sharpener. Provided with education on how to use and dispose of sanitary pads. Education component included puberty education: secondary sex characteristics, biological process of menstruation, and explanation of how pregnancy occurs. Hygiene and menstrual management discussed.
	**Timing of outcome assessment:** 3-months (mid-intervention) and 5-months (conclusion of intervention) post intervention					
	**Outcomes assessed:** self-report questionnaire, School attendance records. Girls’ menstrual calendars. *School attendance records* (% days attended). *Psychosocial outcomes*: Shame, lack of self-confidence, insecurity, difficulty concentrating					
**Hardware Interventions**						
**Oster 2010 [49]**	iRCT	Nepal/Urban and peri-urban schools	198 females aged 12–16 (87% post-menarche)	Provision of menstrual cup and instructions on how to use it	15 months	Girls and their mothers in the intervention group were provided with a menstrual cup (MoonCup) and instructions on how to use the cup were provided by a nurse.
	**Timing of outcome assessment:** 15-months throughout the intervention					
	**Outcomes assessed:** School attendance records. Self-reported attendance, and self-recorded menstrual calendars. *School attendance*: Overall attendance, attendance during menstrual and non-menstrual days.					
**Wilson 2014 [32,33]**	NRS. Non-randomised cluster control trial	Kenya/Non-urban primary and secondary schools	302 post-menarche females aged 11–26 (mean 16 years)	Provision of a training session and materials to make reusable sanitary pads	1 session	A training session on how to make a reusable sanitary pad and provision of enough equipment to make three pads. Girls were given a printed hand-out, adapted from the original pad pattern, to remind them how to make the pad. Instructions about washing and drying and information on the risk of infection or irritation of a damp or poorly washed pad was included. (The workshop did not include general menstrual health education)
	**Timing of outcome assessment:** 1-month after initial training					
	**Outcomes assessed:** self-report questionnaire: *School attendance*: self-reported. *Menstrual hygiene practices*: including use of pads, adequate washing					

iRCT: individually randomised controlled trial, cRCT: cluster randomised controlled trial, NRS: non-randomised study

^1^All interventions were compared to no-treatment controls

Limited information was provided regarding the content of education interventions, but all papers reported including key information such as the origin of menstrual bleeding and menstrual management. The amount of education varied, as did the timing of outcome assessment. The trial by Djalalinia and colleagues [48] in particular failed to describe education content or duration. The three hardware intervention trials differed in the products tested. One provided disposable sanitary pads,[18] one self-made re-usable pads,[32] and one reusable menstrual cups.[49] All trials compared intervention groups to no-intervention controls.

The timing of outcome assessment varied, from immediately after a 120-minute intervention to two-years post intervention. Primary psychosocial outcomes were only assessed in one study.[18,21] Most software trials reported only secondary outcomes of attitude towards menstruation and menstrual knowledge. One study [45] used a previously validated menstrual attitude questionnaire (MAQ; see [50]), however, this did not capture vital psychosocial constructs reflected in background literature. Knowledge of menstruation was assessed via study-specific questionnaires, for which comparability is unclear. Whilst Fakhri and colleagues [46] assessed many relevant outcomes, including self-reported school attendance, authors reported only a single collapsed ‘menstrual health’ variable and did not report menstrual attitude outcomes according to intervention or control group.

### Ongoing studies

Two eligible ongoing study were identified, see S1 Table.

### Risk of bias in included studies

**Fig 2:**
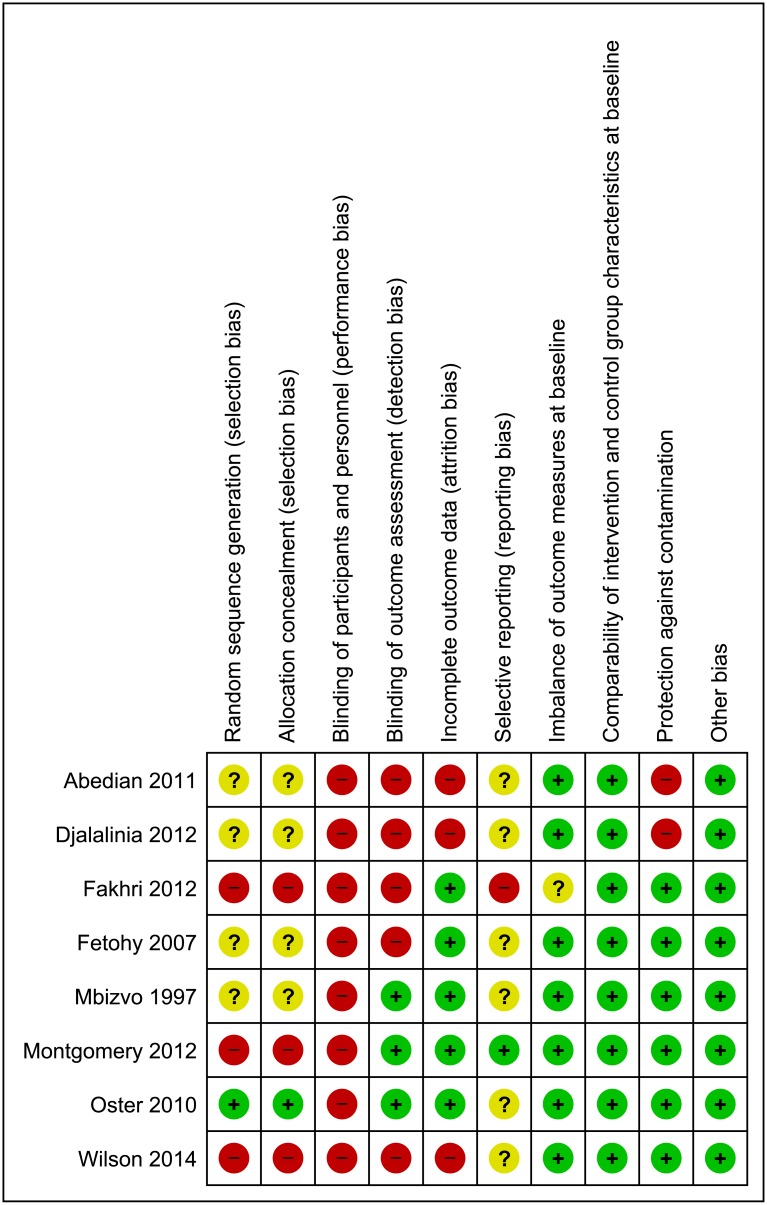
Review authors’ judgements about methodological items for each included study.

Risk of bias assessments for included studies are summarised in Fig 2, and discussed below. Justification for included study risk of bias ratings are reported in S2 Table.

Three studies were non-randomised, representing a high risk of selection bias.[38] Positively, these studies assessed and reported good comparability of intervention and control group characteristics at baseline. Wilson and colleagues [32,33] reported that all outcomes were comparable at baseline. The primary outcome of school attendance was balanced at baseline in the study by Montgomery and colleagues [18], however, psychosocial outcomes were not balanced, representing a high risk for these outcomes. Of randomised studies, only one [49] adequately reported methods of randomisation and allocation concealment. Of the three individually allocated studies, two were vulnerable to contamination.[45,48] Education provided to girls in the same university dormitories or school could have easily been passed to friends in the control condition.

All studies had a high risk of bias due to blinding, as it would not be possible to conceal the intervention from those providing or receiving education or sanitary products. Similarly, the majority of outcomes were self-reported by participants and most included items (e.g., attitudes) vulnerable to self-report biases, and may have been influenced by the lack of blinding. Two studies [18,49] were considered to have a low risk of detection bias as they used official school attendance records. Whilst attendance data may not be highly reliable in the context of LMICs, both studies reported triangulating school records with researcher visits [18] or girls’ own attendance diaries [49] and noted a high level of attendance record reliability. It should be noted that for attitude items reported in Dolan et al.[18,21] a high risk of detection bias is present. Mbvizo and colleagues (1997) tested menstrual knowledge by assessing students’ answers to questions about menstruation; judged to represent low risk of bias as the ability to answer accurately was unlikely to be altered by lack of blinding. There was considerable attrition in Wilson et al.[32,33] as authors were unable to follow-up one intervention school. Djalalinia and colleagues [48] stated in text that 5% of the sample was lost to follow-up, however reported N’s deviate from this figure with 1231 participants of 1823 (67.5%) reported on at two-years post intervention, with even smaller numbers displayed in results tables. Abedian et al.[45] reported modest attrition, but failed to employ an intention-to-treat analysis. Fetohy [47] assessed outcomes immediately after the education session, resulting in no attrition. Despite a 15-month duration, Oster and Thornton [49] reported negligible drop out (one participant), and employed an intention-to-treat analysis. Some missing outcome data was reported by Montgomery et al.[18,21] but this was judged to be low risk in light of analytic approaches, including imputing missing values. Only one study reported trial registration.[18] Of the remaining studies, five reported all outcomes included in methods. One study,[46] reported only an aggregated outcome, and one [44] did not include a list of measured outcomes in methods.

No other sources of bias were identified, although the suitability of outcome measures and study quality varied and are highlighted further in discussion.

### Effects of interventions: software interventions

Results of software intervention trials are summarised in Table 3.

**Table 3:** Effects of software interventions.

Study ID	Design	Age, N per condition	Intervention^1^	Eligible Outcome/s	Measure of Effect SMD^2^ (95%CI)	Adjustments
**Abedian 2011 [45]**	iRCT	19–25 years. 104 intervention, 61 control	Peer-led and health-provider-led ‘self-care’ education (collapsed)	Menstrual knowledge	2.29 (1.88, 2.69)	No adjustment
				*Menstrual Attitude*:		No adjustment
				Menstruation as debilitating	-1.26 (-1.61, -0.92)	
				Menstruation as bothersome	-0.40 (-0.72, -0.08)	
				Menstruation as natural	0.66 (0.34, 0.99)	
				Prediction of onset of menstruation	0.22 (-0.10, 0.53)	
				Denial of effect of menstruation	0.76 (0.43, 1.08)	
**Djalalinia 2012 [48]**	iRCT	11–15 years. Total 1823 (intervention and control N’s not reported)	Parent-led or school health trainer-led menstruation education (collapsed)	Feelings at menarche (confusion, scared, uncomfortable feeling, good feeling)	Unable to calculate measures of effect. N’s and percentages not adequately reported in study table and text. Authors report control participants were more likely to experience negative feelings at menstruation (confusion, being scared, feeling uncomfortable) than those in the health trainers groups (p < .001).	No adjustment
				Hygiene practices	The trained groups were more likely to take appropriate actions at menarche than controls but this was not significant. Authors report continuing to exercise during menstruation was most common amongst the health trainers group (p < .05)	No adjustment
**Fakhri 2012 [46]**	Cluster Controlled before after	14–18 years. 349 intervention, 349 control	Youth and School Health Department-run puberty and menstrual health education	Menstrual health (collapsed outcome) (Risk ratio of good or excellent ‘menstrual health’ in contrast to ‘average’ or ‘poor’ menstrual health).	RR = 1.30 (1.04, 1.64)^3^ Control RR = 1.00	No adjustment
				Usual bathing during menstruation	RR = 1.29 (1.10, 1.51) ^3^ Control RR = 1.00	
				Attitude toward menstruation	Not reported by intervention and control group (data requested, not provided)	
**Fetohy 2007 [47]**	cRCT	14–17 years. 124 intervention,124 control	Menstrual education sessions	Menstrual knowledge	2.23 (1.98, 2.62)	No adjustment
				Menstrual attitude	1.82 (1.52, 2.11)	
				Menstrual practices	0.97 (0.70, 1.23)	
**Mbizvo 1997 [44]**	cRCT	10–19 years. 1159 intervention, 530 control (51% female)	Puberty education through posters and pamphlets	Menstrual knowledge	No comparable outcomes. Outcomes reported collapsed for males and females (authors unable to provide stratified data)	
**Montgomery 2012 [18,21]**	NRS cluster control trial	12–18 years. 25 education only intervention, 35 control	Puberty education alone condition	School attendance	0.63 (0.11, 1.16)	No adjustment
				*Psychosocial outcomes*: *s*hame, lack of self-confidence, insecurity, difficulty concentrating	Dichotomous outcomes. Risk ratios could not be calculated. Clusters varied widely at baseline. Control group psychosocial outcomes were not assessed at follow-up and test-retest reliability of measures was not reported/evaluated (therefore difference scores could not be compared). Authors reported pre-post analysis for each cluster. Authors reported psychosocial outcomes of shame, self-confidence, insecurity and difficulty concentrating did not improve in the education-alone condition	No adjustment

^1^All interventions were compared to no-treatment controls

^2^ Standardised Mean Difference (Cohen’s *d*) calculated from means and standard deviations and data provided in text (for guidance; small 0.2, medium 0.5, and large 0.8 effects)

^3^RR = Risk Ratio, calculated from reported study frequencies for dichotomous outcomes.

#### Education

Only one study assessed the impact of education on school attendance.[18] When comparing the education-only school (n = 25) to the control school (n = 35) a medium effect (SMD = 0.63 95%CI 0.11–1.16) was found. Although a broad confidence interval, and comparison of single clusters cautions against over-interpretation.[39]

No studies assessed secondary education measures of achievement, or engagement.

#### Employment

No studies assessed the impact of MHM interventions on employment.

#### Psychosocial outcomes

Primary psychosocial outcomes were assessed in one study.[18,21] Authors reported that outcomes of shame, lack of self-confidence, insecurity, and difficulty concentrating did not improve in the education-only arm of the study.[21]

Djalalinia and colleagues [48] evaluated primary outcomes of fear and confusion specific to menarche. Authors reported that negative psychological effects of menarche were lower in the groups which received the intervention. However, insufficient data were reported to calculate an effect size, and findings should be interpreted with caution in light of unclear attrition and analyses.

Most software trials reported only secondary outcomes of attitude towards menstruation, or menstrual knowledge and practices (Table 3). Abedian et al.[45] found a large positive impact of education on menstrual knowledge. On average, scores on the 10-item test designed by study authors increased from 3.86 to 8.99. The study used the MAQ (Table 2) to assess attitudes and found medium to large effects on most sub-scales (Table 3). Fetohy [47] found a large positive effect of education on menstrual attitudes compared to controls, however menstrual attitude was conceptualised an attitude towards healthy practices which fails to capture attitudes towards experiencing menstruation and is not comparable to other studies. Fetohy [47] found a large difference in menstrual knowledge post-education, with intervention and control groups differing by approximately 10-points on a test scored from 0 to 33. Similarly there was a large standardised mean difference between intervention and control students in self-reported hygiene behaviours.[47] However, this outcome was measured immediately after a single session of education, leaving students no time to have changed behaviours. Thus this result is likely to represent recall bias or social desirability effects, and demonstrates the biases inherent in self-reported behavioural outcomes, particularly where blinding to condition is not possible. Fakhri and colleagues [46] found a significant impact of their intervention on the inappropriately collapsed ‘menstrual health’ variable which included a wide range of practices.

### Effects of interventions: hardware interventions

#### Education

**Fig 3:**
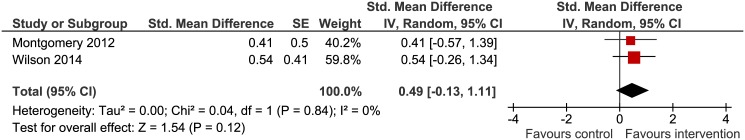
Hardware intervention compared with no treatment on school attendance.

School attendance was assessed in all three trials. In accordance with review protocol, randomised studies [49] were not combined with non-randomised studies. Thus two studies [18,32] were eligible for meta-analysis. Intervention heterogeneity was high as studies differed in the products provided and education was included alongside pads in one study.[18] Random-effects meta-analysis with standard-errors corrected for clustering revealed a moderate non-significant pooled effect (SMD = 0.49, 95%CI -0.13, 1.11), see Fig 3. Statistical heterogeneity was negligible (Chi^2^ = 0.04, *df* = 1, *p* = 0.84, I^2^ = 0%).

Oster and Thornton [49] reported education outcomes using days rather than participants as the unit of analysis. For a 180-day school-year, authors report that provision of a menstrual cup resulted in a small but non-significant improvement (β = 0.14, *p*>.05). The interaction between receipt of a menstrual cup and days of school missed during menstrual periods was investigated and also found to be a small non-significant effect (β = 0.02, *p*>.05).

#### Psychosocial outcomes

One study [18,21] reported on psychosocial outcomes. Psychosocial outcomes varied significantly between clusters at baseline. In addition, these outcomes were not evaluated in the control cluster at follow-up, and test-retest reliability of scales was not reported (or evaluated). Dolan et al.[21] were only able to report pre-post intervention changes in psychosocial outcomes for each cluster. Authors reported that for both sites where pads and education were provided, shame, lack of confidence, insecurity, and difficulty concentrating were improved following the intervention, with many outcomes (including shame, insecurity and difficulty concentrating) reported by approximately 25% fewer girls in each site at follow-up.[21] These findings represent a much higher risk of bias as they were not compared to a control group, and it may be the case that negative psychological consequences of menstruation decrease naturally over time.

## Discussion

This review identified six software, and three hardware trials of MHM interventions. Whilst heterogeneous with regard to intervention, context and delivery, software interventions were generally found to improve knowledge of menstruation.[45,46] Menstrual knowledge is hypothesised to improve girls’ MHM and reduce negative psychosocial consequences.[17,28–30] Some supporting evidence for these outcomes was found, with management practices reported to have been improved by education in three studies.[46–48] However, unclear measures and self-report biases mean results should be interpreted with caution. In terms of psychosocial outcomes, more positive attitudes towards menstruation were found for girls who had received education in two studies.[45,47] One study [45] used a previously validated menstrual attitude questionnaire (MAQ;[50]). However, this measure was developed in a high-income, US-context. Survey items, for example, ‘*menstruation is an obvious example of the rhythmicity which pervades all of life’* and ‘*a woman who attributes her irritability to her approaching menstrual period is neurotic’* [50] do not reflect the fear and stigma facing women in LMICs.[10,11] Only two studies measured primary psychosocial outcomes. One RCT found a positive influence of education on feelings at menarche,[48] in contrast to a non-randomised study [18,21] which found no improvements in shame, self-confidence, or insecurity following education. This same software intervention found positive effects on school attendance.[18] However, the non-randomised pilot study was small, with single clusters compared.

School attendance was assessed in all three hardware trials. Moderate, but non-significant, improvements in school attendance were found for hardware interventions providing varied sanitary products. One iRCT found no significant impact of providing menstrual cups.[49] Although context should be taken into account, with very high attendance reported at baseline a lack of results may reflect a ceiling effect. Further accurate uptake of menstrual cups may have been difficult to capture as these could be difficult to use for young girls. Meta-analysis of two non-randomised studies providing disposable sanitary pads [18] and reusable home-made pads [32] revealed a moderate non-significant effect. Given the small sample and cluster sizes of the studies, both pilots, this may be due to a lack of power. Larger trials are needed to determine the true effectiveness of MHM hardware interventions. In such trials, assessment of supplementary outcomes such as achievement or engagement would triangulate findings. Only one study [18,21] assessed hardware intervention impact on psychological outcomes, with reductions in shame, lack of confidence, insecurity, and difficulty concentrating reported. However, as noted previously only pre-post analysis of individual clusters was possible.

High risk of bias was present in the included studies. Three non-randomised studies were included. Positively, appraisal using additional items suggested by the EPOC group [39] showed intervention and control groups in non-randomised studies to be comparable at baseline on characteristics assessed. Risk of contamination was an issue in two individually randomised studies.[45,48] Studies suffered from inadequate reporting with many items in risk of bias assessments *‘*unclear’. Further, few studies reported implementation, or analytic strategies, for appraisal.

A lack of appropriate outcome assessment limits the conclusions which can be drawn currently. No software trials assessed academic outcomes, and only two studies [18,21,48] assessed primary psychosocial outcomes. Whilst such outcome measurement is challenging in LMIC contexts, no studies evaluated potential harms.[37] Stigma, harassment, and unwanted sexual attention arising from the disclosure of menstrual status have been documented.[1,16,17] The identification of menstruating females through interventions may ‘out’ girls’ menstrual status, resulting in negative effects on attendance or psychological harm.

This review has highlighted many weaknesses in the existing body of evidence for MHM interventions. The challenges of conducting rigorous evaluations in LMICs should not be overlooked when considering the value of these findings. Furthermore, large gaps in MHM research more broadly contribute to the difficulty of conducting intervention trials. As noted, the lack of measures specific to MHM limits the reliability of outcome measures. Similarly, accurately assessing education outcomes may be challenging due to the differing reliability of school attendance registers or comparability of achievement measures across contexts. Sparse data exists regarding optimal genital cleaning practices for women and girls during menstruation, or the contribution of menstrual absorbent types to genital infection, comfort or education or psychosocial outcomes.[22] Age of menarche differs contextually, and targeting interventions to the correct age group may be difficult in absence of population-specific data.[51–53] Finally, as qualitative research has revealed, menstruation represents a taboo topic for many, and accurately capturing practices, attitudes or disseminating education to communities may fail if strategies lack cultural sensitivity.[12]

The present review comprehensively searched peer-reviewed and grey literature. Searches identified a large number of studies, most of which were of poor quality. All papers included in past reviews were identified. In addition, many excluded studies were not captured in previous reviews [31] although were likely to have met their broader inclusion criteria. Publication bias could not be assessed as the evidence base is still limited. This review did not include interventions to address dysmenorrhoea, painful menses in women with normal pelvic anatomy,[54] which has been identified as an issue affecting attendance at work,[9] school,[13,27,55] and contributing to negative experiences of menstruation.[13,27] However, background searching suggested interventions targeting dysmenorrhoea in LMICs are yet to be trialled.

The use of school attendance, rather than achievement or engagement, as the primary education outcome could be criticised. Authors have argued that a singular focus on attendance is a limitation of present literature.[16,29,56,57] However, no studies reported on academic achievement so this would not have affected the conclusions of the review.

### Implications for future practice and research

There is insufficient evidence to determine the effectiveness of MHM interventions, although some positive indicators are emerging. In seeking to implement programs, practitioners and policy-makers should consider comprehensive evaluations. This review has provided a summary and critical appraisal of interventions and assessment methods used to date, which may aid the development of such evaluations. Larger, randomised trials are needed to determine the impact of MHM interventions. In such trials, cRCTs are likely to be most appropriate for avoiding contamination issues. Trials must compare the various intervention strategies proposed; including both hardware and software interventions and the interaction between them. To aid this process, interventions should be based on a clear theory of change which includes the various individual and contextual factors which contribute to women and girls MHM. Such theories should be tested, and mediators and moderators of effects identified, for example the distribution of reusable sanitary pads may not be effective in the absence of improvements to WASH. Absorbent sustainability, acceptability, comfort, and risk of reproductive tract infections must all be considered when selecting sanitary products appropriate for interventions. A reliable and consistent assessment of all aspects of MHM [1] in observational studies and trials may serve to draw attention to the various aspects of MHM contributing to outcomes.

As noted above, there is a lack of research capturing MHM practices or quantifying the health, education, and psychosocial consequences of different MHM practices. Providing data on the hypothesised causes and consequences of poor MHM would help guide the development and evaluations in future. These trials should seek to use validated measures of psychosocial constructs and measures of women’s wellbeing specific to, or which include, MHM. This would improve comparability across studies, enabling researchers to grasp the severity of distress caused by menstrual poverty, and the influence of interventions.

Studies are needed on the impact of MHM on adult women, and potential WASH interventions as no trials were identified. Future trials must evaluate potential harms of interventions, particularly consequences of ‘outing’ menstruating girls in contexts with high levels of stigma associated with menstruation.

## Supporting Information

S1 Table Characteristics of ongoing studies. (DOCX)

S2 Table Study risk of bias assessments. (DOCX)

S3 Table Full-text studies excluded with reasons. (DOCX)

S4 Table PRISMA Checklist. (DOC)

S1 Text Other publications arising from this work: oral presentation. (DOCX)

## References

[1] Sommer M, Sahin M. Overcoming the Taboo: Advancing the Global Agenda for Menstrual Hygiene Management for Schoolgirls. American journal of public health. 2013;103:1556–1559. 10.2105/AJPH.2013.301374 23865645 PMC3780686

[2] Dasgupta A, Sarkar M. Menstrual hygiene: How hygienic is the adolescent girl? Indian journal of community medicine. 2008;33:77 10.4103/0970-0218.40872 19967028 PMC2784630

[3] Khanna A, Goyal R, Bhawsar R. Menstrual Practices and Reproductive Problems A Study of Adolescent Girls in Rajasthan. Journal of health management. 2005;7:91–107.

[4] El-Gilany AH, Badawi K, El-Fedawy S. Menstrual hygiene among adolescent schoolgirls in Mansoura, Egypt. Reproductive Health Matters. 2005;13:147–152. 10.1016/S0968-8080(05)26191-8 16291496

[5] Adinma ED, Adinma J. Perceptions and practices on menstruation amongst Nigerian secondary school girls. African Journal of Reproductive Health. 2009;12:74–83. 20695158

[6] Lloyd CB, Young J. New lessons: the power of educating adolescent girls: a Girls Count report on adolescent girls. 2009.

[7] Grant M, Lloyd C, Mensch B. Menstruation and School Absenteeism: Evidence from Rural Malawi. Comparative Education Review. 2013;57:260–284. 25580018 10.1086/669121 PMC4286891

[8] Business for Social Responsibility (BSR). HERproject: Health Enables Returns The Business Returns from Women’s Health Programs. 2011.

[9] Business for Social Responsibility (BSR). HERproject Investing in Women for a Better World. 2012.

[10] Sommer M. Ideologies of sexuality, menstruation and risk: girls' experiences of puberty and schooling in northern Tanzania. Culture, health & sexuality. 2009;11:383–398. 10.1080/13691050902722372 19326264

[11] Crichton J, Okal J, Kabiru CW, Zulu EM. Emotional and Psychosocial Aspects of Menstrual Poverty in Resource-Poor Settings: A Qualitative Study of the Experiences of Adolescent Girls in an Informal Settlement in Nairobi. Health Care for Women International 2013;34:891–916. 10.1080/07399332.2012.740112 23570366

[12] Sommer M, Ackatia-Armah N, Connolly S, Smiles D. A comparison of the menstruation and education experiences of girls in Tanzania, Ghana, Cambodia and Ethiopia. Compare: A Journal of Comparative and International Education. 2014:45(4):589–609.

[13] Ali TS, Rizvi SN. Menstrual knowledge and practices of female adolescents in urban Karachi, Pakistan. Journal of adolescence. 2010;33:531–541. 10.1016/j.adolescence.2009.05.013 19589587

[14] Kumar A, Srivastava K. Cultural and social practices regarding menstruation among adolescent girls. Social work in public health. 2011;26:594–604. 10.1080/19371918.2010.525144 21932979

[15] Deo D, Ghattargi C. Perceptions and practices regarding menstruation: a comparative study in urban and rural adolescent girls. Indian J Community Med. 2005;30(1):33.

[16] Pillitteri SP. School menstrual hygiene management in Malawi: more than toilets. WaterAid report. 2011.

[17] House S, Mahon T, Cavill S. Menstrual hygiene matters: a resource for improving menstrual hygiene around the world. WaterAid. 2012.

[18] Montgomery P, Ryus CR, Dolan CS, Dopson S, Scott L. Sanitary Pad Interventions for Girls' Education in Ghana: A Pilot Study. PloS one. 2012;7:e48274 10.1371/journal.pone.0048274 23118968 PMC3485220

[19] Crofts T, Fisher J. Menstrual hygiene in Ugandan schools: an investigation of low-cost sanitary pads. Journal of Water, Sanitation and Hygiene for Development. 2012;2:50–58.

[20] Garg R, Goyal S, Gupta S. India moves towards menstrual hygiene: subsidized sanitary napkins for rural adolescent girls—issues and challenges. Maternal and child health journal. 2012;16:767–774. 10.1007/s10995-011-0798-5 21505773

[21] Dolan CS, Ryus CR, Dopson S, Montgomery P, Scott L. A blind spot in girls education: menarche and its webs of exclusion in Ghana. Journal of International Development. 2013;26:643–657.

[22] Das P, Baker KK, Dutta A, Swain T, Sahoo S, Das BS, et al Menstrual Hygiene Practices, WASH Access and the Risk of Urogenital Infection in Women from Odisha, India. PloS one. 2015;10:e0130777 10.1371/journal.pone.0130777 26125184 PMC4488331

[23] Fisher J. For her it's the big issue: putting women at the centre of water supply, sanitation and hygiene. Water, Sanitation and Hygiene Evidence Report. 2006.

[24] Abrahams N, Mathews S, Ramela P. Intersections of ‘sanitation, sexual coercion and girls’ safety in schools’. Tropical Medicine & International Health. 2006;11:751–756. 16640629 10.1111/j.1365-3156.2006.01600.x

[25] Mahon T, Fernandes M. Menstrual hygiene in South Asia: a neglected issue for WASH (water, sanitation and hygiene) programmes. Gender & Development. 2010;18:99–113.

[26] Chothe V, Khubchandani J, Seabert D, Asalkar M, Rakshe S, Firke A, et al Students’ Perceptions and Doubts About Menstruation in Developing Countries A Case Study From India. Health promotion practice. 2014;15(3):319–326. 10.1177/1524839914525175 24618653

[27] Santina T, Wehbe N, Ziade F. Exploring dysmenorrhoea and menstrual experiences among Lebanese female adolescents. Eastern Mediterranean Health Journal. 2012;18(8):1. 10.26719/2012.18.8.857 23057375

[28] Kirk J, Sommer M. Menstruation and body awareness: linking girls’ health with girls’ education. Royal Tropical Institute (KIT), Special on Gender and Health. 2006;1–22.

[29] McMahon SA, Winch PJ, Caruso BA, Obure AF, Ogutu EA, Ochari IA, et al 'The girl with her period is the one to hang her head' Reflections on menstrual management among schoolgirls in rural Kenya. BMC international health and human rights. 2011;11: 7 10.1186/1472-698X-11-7 21679414 PMC3129305

[30] Sommer M. An Early Window of Opportunity for Promoting Girls' Health: Policy Implications of the Girl's Puberty Book Project in Tanzania. International Electronic Journal of Health Education. 2011;14: 77–92.

[31] Sumpter C, Torondel B. A Systematic Review of the Health and Social Effects of Menstrual Hygiene Management. PloS one. 2013;8:e62004 10.1371/journal.pone.0062004 23637945 PMC3637379

[32] Wilson E, Reeve J, Pitt A. Education. Period. Developing an acceptable and replicable menstrual hygiene intervention. Development in Practice. 2014;24:63–80.

[33] Wilson EF, Reeve JMK, Pitt AH, Sully BG, Julious SA. INSPIRES: Investigating a reusable sanitary pad intervention in a rural educational setting—evaluating the acceptability and short term effect of teaching Kenyan school girls to make reusable sanitary towels on absenteeism and other daily activities: a partial preference parallel group, cluster randomised control trial. Research Report ScHARR Report Series (27) School of Health and Related Research, University of Sheffield. 2012. Available: http://eprints.whiterose.ac.uk/43906/1/Irise_report_-_Dec_2012_%5BSAJ%5D_v2_(1).pdf

[34] Birdthistle I, Dickson K, Freeman M, Javidi L. What Impact Does the Provision of Separate Toilets for Girls at Schools Have on Their Primary and Secondary School Enrolment, Attendance and Completion?: A Systematic Review of the Evidence: EPPI-Centre, Social Science Research Unit, Institute of Education, University of London. 2011.

[35] Jasper C, Le TT, Bartram J. Water and sanitation in schools: a systematic review of the health and educational outcomes. International journal of environmental research and public health. 2012;9:2772–2787. 10.3390/ijerph9082772 23066396 PMC3447586

[36] Ministry of Health & Family Welfare, Government of India. Two year (2009–2011) achievements of Ministry of Health & Family Welfare unveiled. 2012. Available: http://pib.nic.in/newsite/erelease.aspx?relid=72307.

[37] Moher D, Liberati A, Tetzlaff J, Altman DG. Preferred reporting items for systematic reviews and meta-analyses: the PRISMA statement. Annals of internal medicine. 2009;151:264–269. 19622511 10.7326/0003-4819-151-4-200908180-00135

[38] Higgins JPT, Green S. Cochrane Handbook for Systematic Reviews of Interventions Version 5.1.0 [updated March 2011]. The Cochrane Collaboration 2011 Available from: www.cochrane-handbook.org.

[39] Effective Practice and Organisation of Care Group (EPOC). EPOC Resources for review authors. Oslo: Norwegian Knowledge Centre for the Health Services 2013 Available: http://epocoslo.cochrane.org/epoc-specific-resources-review-authors.

[40] World Bank. Country and Lending Groups: Data. 2014. Available: http://data.worldbank.org/about/country-classifications.

[41] Adams J, World Health Organisation. Water, sanitation and hygiene standards for schools in low-cost settings: World Health Organization Geneva, Switzerland 2009.

[42] Inter-agency Task Force on Gender and Water (GWTF). Gender, water and sanitation: A Policy Brief. New York: United Nations 2006.

[43] Review Manager (RevMan) [Computer program] RMRC. Version 5.3 Copenhagen: The Nordic Cochrane Centre, The Cochrane Collaboration 2012.

[44] Mbizvo M, Kasule J, Gupta V, Rusakaniko S, Kinoti S, Mpanju-Shumbushu W, et al Effects of a randomized health education intervention on aspects of peproductive health knowledge and reported behaviour among adolescents in Zimbabwe. Social Science & Medicine. 1997;44: 573–577. 9032825 10.1016/s0277-9536(96)00204-3

[45] Abedian Z, Kabirian M, Mazlom SR, Mahram B. The effects of peer education on health behaviors in girls with dysmenorrhea. Journal of American Science. 2011;7:1.

[46] Fakhri M, Hamzehgardeshi Z, Golchin NAH, Komili A. Promoting menstrual health among Persian adolescent girls from low socioeconomic backgrounds: a quasi-experimental study. BMC public health. 2012;12:193 10.1186/1471-2458-12-193 22420743 PMC3348061

[47] Fetohy EM. Impact of a health education program for secondary school Saudi girls about menstruation at Riyadh city. J Egypt Public Health Assoc. 2007;82:105–126. 18217327

[48] Djalalinia S, Tehrani FR, Afzali HM, Hejazi F, Peykari N. Parents or School Health Trainers, which of them is Appropriate for Menstrual Health Education? International journal of preventive medicine. 2012;3:622 23024851 PMC3445278

[49] Oster E, Thornton R. Menstruation, sanitary products, and school attendance: Evidence from a randomized evaluation. American Economic Journal: Applied Economics. 2011;3(1):91–100

[50] Brooks-Gunn J, Ruble DN. The menstrual attitude questionnaire. Psychosomatic Medicine. 1980;42:503–512. 7465737 10.1097/00006842-198009000-00005

[51] Anderson SE, Dallal GE, Must A. Relative weight and race influence average age at menarche: results from two nationally representative surveys of US girls studied 25 years apart. Pediatrics. 2003;111:844–850. 12671122 10.1542/peds.111.4.844

[52] Parent AS, Teilmann G, Juul A, Skakkebaek NE, Toppari J, Bourguignon JP. The timing of normal puberty and the age limits of sexual precocity: variations around the world, secular trends, and changes after migration. Endocrine reviews. 2003;24:668–693. 14570750 10.1210/er.2002-0019

[53] Sommer M, Mmari K. Addressing Structural and Environmental Factors for Adolescent Sexual and Reproductive Health in Low-and Middle-Income Countries. American Journal of Public Health. 2015;e1–e9. 10.2105/AJPH.2015.302740 PMC4566559 26270290

[54] Avasarala AK, Panchangam S. Dysmenorrhoea in different settings: Are the rural and urban adolescent girls perceiving and managing the dysmenorrhoea problem differently? Indian journal of community medicine. 2008;33:246 10.4103/0970-0218.43231 19876499 PMC2763707

[55] Tavallaee M, Joffres MR, Corber SJ, Bayanzadeh M, Rad MM. The prevalence of menstrual pain and associated risk factors among Iranian women. Journal of Obstetrics and Gynaecology Research. 2011;37:442–451. 10.1111/j.1447-0756.2010.01362.x 21208343

[56] Sommer M. Where the education system and women's bodies collide: The social and health impact of girls' experiences of menstruation and schooling in Tanzania. Journal of Adolescence. 2010;33:521–529. 10.1016/j.adolescence.2009.03.008 19395018

[57] Haver J, Caruso BA, Ellis A, Sahin M, Villasenor JM, Andes KL, et al ‘WASH in Schools Empowers Girls’ Education in Masbate Province and Metro Manila, Philippines: An assessment of menstrual hygiene management in schools’. New York: UNICEF, Emory University 2013.

