# Supplementary material for: Do Menstrual Hygiene Management Interventions Improve Education and Psychosocial Outcomes for Women and Girls in Low and Middle Income Countries? A Systematic Review
Source: PLoS One. 2016 Feb 10;11(2):e0146985. doi: 10.1371/journal.pone.0146985 (PMC4749306; doi:10.1371/journal.pone.0146985)
Supplement: S1 Table — (DOCX) [file pone.0146985.s001.docx]

**S1 Table**

**Characteristics of ongoing studies**

| **Montgomery et al., 2011.** | |
| --- | --- |
| Trial registration: PACTR201503001044408 | |
| Trial name or title | Menstruation and the Cycle of Poverty |
| Methods | **Design:** cluster randomised controlled trial  **Study Objective:** To evaluate the effectiveness of sanitary pad provision and puberty education on girls’ school attendance and psychosocial outcomes  **Location:** Kamuli District, Uganda  **Setting:** Non-urban primary and secondary schools  **N:** 560 schoolgirls |
| Participants | **Age:** 12-15  **Exclusions:** post-menarche females |
| Interventions | **Comparison:** No intervention  **Intervention:** Four conditions will be tested among menstruating schoolgirls aged 12-15: pads alone, education alone, education-plus-pads, and a control group. |
| Outcomes | **Form of data collection:** Self-report questionnaire, school attendance records  **Outcomes assessed:** *School attendance* % days attended  *Psychosocial outcomes:* self-reported feelings of shame, lack of self-confidence, insecurity, difficulty concentrating |
| Starting date | September, 2011 |
| Progress | Trial completed, awaiting publication. |
| **Phillips-Howard et al., 2014.** | |
| Trial registration: ISRCTN17486946 | |
| Trial name or title | Menstrual Solutions Study |
| Methods | **Design:** cluster randomised controlled trial  **Study Objective:** To determine the acceptability, use and safety of menstrual cups compared with other menstrual products  **Location:** Western Kenya  **Setting:** Schoolgirls in rural primary schools in western Kenya, living in a demographic health and surveillance system site |
| Participants | **Age:** 14-16  **Exclusions:** post-menarche females |
| Interventions | **Comparison:** No intervention  **Intervention:** Two conditions will be tested: menstrual cups and disposable sanitary pads. All participants are given soap for hand-washing. |
| Outcomes | **Form of data collection:** Self-report questionnaire, interviews  **Outcomes assessed:** Outcomes include: cultural acceptance, use, satisfaction, costs and safety of menstrual cups, compared with sanitary pads and ‘usual practice’, and assesses school, sexual and reproductive health, and wellbeing. |
| Starting date | March, 2012 |
| Progress | Trial completed, awaiting publication. |
