# Supplementary material for: Do Menstrual Hygiene Management Interventions Improve Education and Psychosocial Outcomes for Women and Girls in Low and Middle Income Countries? A Systematic Review
Source: PLoS One. 2016 Feb 10;11(2):e0146985. doi: 10.1371/journal.pone.0146985 (PMC4749306; doi:10.1371/journal.pone.0146985)
Supplement: S2 Table — (DOCX) [file pone.0146985.s002.docx]

**S2 Table**

**Study risk of bias assessments**

| ***Abedian 2011*** | | |
| --- | --- | --- |
| **Bias** | **Author’s judgement** | **Support for judgement** |
| Random sequence generation (selection bias) | Unclear Risk | “dysmenorrheic girls from three different dormitories of Ferdowsi University fulfilled the inclusion criteria and were randomly divided into three groups” p.432 |
| Allocation concealment (selection bias) | Unclear Risk | Not reported |
| Blinding of participants and personnel (performance bias)  All outcomes | High Risk | “dysmenorrheic self-care education sessions were carried out by a midwife in the health-provider-led self-care education group and by peer educators in the peer-led self-care education group with the method of small-group discussion” p. 432 |
| Blinding of outcome assessment (detection bias)  All outcomes | High Risk | All outcomes self-reported. Whilst menstrual knowledge not likely to be affected by lack of blinding, attitude towards menstruation high risk of bias. |
| Incomplete outcome data (attrition bias)  All outcomes | High Risk | “The recruited sample comprised a total of 209 dysmenorrheic girls. Among them, 14 clients were selected as peer educators and 30 clients were excluded from the study, constituting a drop-out rate of 15.3%.” p. 433  Intention-to-treat analysis not used. |
| Selective reporting (reporting bias) | Unclear Risk | No trial registration number given.  All outcomes included in methods reported. |
| Imbalance of outcome measures at baseline | Low Risk | Individually randomised trial. No differences at baseline. |
| Comparability of intervention and control group characteristics at baseline | Low Risk | Individually randomised trial. Group characteristics comparable at baseline. |
| Protection against contamination | High Risk | None. Study included females living in university dormitories. Control group may have been exposed to information presented in the intervention from peers receiving the intervention. |
| Other bias | Low Risk | N/A |

***Djalalinia 2012***

| **Bias** | **Author’s judgement** | **Support for judgement** |
| --- | --- | --- |
| Random sequence generation (selection bias) | Unclear Risk | Method of randomisation not reported  “At first we conducted a survey on the menstrual health of the participants. After that the participants were randomly divided into three groups” |
| Allocation concealment (selection bias) | Unclear Risk | Not reported |
| Blinding of participants and personnel (performance bias)  All outcomes | High Risk | “The second group was trained by the school health trainers, who were themselves trained by project experts; and the third group received designed training through their parents.” |
| Blinding of outcome assessment (detection bias)  All outcomes | High Risk | All outcomes self-reported. Feelings towards menstruation at menarche high risk of bias as participants not blinded to intervention. |
| Incomplete outcome data (attrition bias)  All outcomes | High Risk | Attrition unclear with conflicting reports.  “From 15 Middle Schools in Tehran, through quota random sampling, 1823 female students were selected proportionally and allocated randomly to three groups” p. 1  “In final evaluation… we evaluated about a 5% loss to follow-up”… “overall among the final completed questionnaires, the response rate was 95%” p. 3  “Following a two-year intervention, information on 1231 female adolescents was included for assessment” p.3  N’s provided in tables inconsistent with 95% or 67.5% (1231/1823) response rate. |
| Selective reporting (reporting bias) | Unclear Risk | No trial registration number given.  All outcomes included in methods reported. |
| Imbalance of outcome measures at baseline | Low Risk | Individually randomised trial. |
| Comparability of intervention and control group characteristics at baseline | Low Risk | Individually randomised trial. Characteristics comparable at baseline. |
| Protection against contamination | High Risk | None. Study included students in the same schools. Control group may have been exposed to information presented in the intervention from peers receiving the intervention. |
| Other bias | Low Risk | NA |

***Fakhri 2012***

| **Bias** | **Author’s judgement** | **Support for judgement** |
| --- | --- | --- |
| Random sequence generation (selection bias) | High Risk | Non randomised.  “The health promotion initiative by the education organisation in Mazandaran province, in north of Iran, was conducted in selected high schools... The control group comprised high school students in the Mazandaran province who did not participate in the education”  “As much as possible, the control samples were selected from the nearest neighbour school”  p. 193 |
| Allocation concealment (selection bias) | High Risk | Non randomised. |
| Blinding of participants and personnel (performance bias)  All outcomes | High Risk | “The health promotion initiative by the education organisation in Mazandaran province, in north of Iran, was conducted in selected high schools. The project included 10 2hr educational sessions using adolescent health resources”  Students and providers aware of intervention |
| Blinding of outcome assessment (detection bias)  All outcomes | High Risk | All outcomes self-reported. Included outcomes of self-reported menstrual attitude and practices high risk of bias due to lack of blinding. |
| Incomplete outcome data (attrition bias)  All outcomes | Low Risk | No missing outcome data |
| Selective reporting (reporting bias) | High Risk | No trial registration number given.  Items listed in methods not reported in results or outcomes reported collapsed across many measures inappropriately. |
| Imbalance of outcome measures at baseline | Unclear Risk | Outcome measures not assessed at baseline |
| Comparability of intervention and control group characteristics at baseline | Low Risk | “As must as possible, the control samples were selected from the nearest neighbour school. Factors matched in the study and control group were school types (rural or urban), grade, age, and educational field of study.” p. 194 |
| Protection against contamination | Low Risk | Cluster design. “The health promotion initiative by the education organisation in Mazandaran province, in north of Iran, was conducted in selected high schools.” p.194. |
| Other bias | Low Risk | N/A |

***Fetohy 2007***

| **Bias** | **Author’s judgement** | **Support for judgement** |
| --- | --- | --- |
| Random sequence generation (selection bias) | Unclear Risk | No information on randomisation process reported |
| Allocation concealment (selection bias) | Unclear Risk | Not reported |
| Blinding of participants and personnel (performance bias)  All outcomes | High Risk | “The session of the program started after break-time for the intervention classes (the school’s nurse and two social workers collected the students in the mosque of the school) and the session took 3 usual class times (120 minutes).” |
| Blinding of outcome assessment (detection bias)  All outcomes | High Risk | All outcomes self-reported. Whilst menstrual knowledge less likely to be impacted by lack of blinding, self-reported menstrual attitudes likely to be influenced by lack of blinding. |
| Incomplete outcome data (attrition bias)  All outcomes | Low Risk | No missing outcome data |
| Selective reporting (reporting bias) | Unclear Risk | No trial registration number given. All outcomes included in methods reported. |
| Imbalance of outcome measures at baseline | Low Risk | Randomised design |
| Comparability of intervention and control group characteristics at baseline | Low Risk | Randomised design. Participant characteristics comparable at baseline. |
| Protection against contamination | Low Risk | Cluster randomised design. |
| Other bias | Low Risk | N/A |

***Mbizvo 1997***

| **Bias** | **Author’s judgement** | **Support for judgement** |
| --- | --- | --- |
| Random sequence generation (selection bias) | Unclear Risk | Method of randomisation not reported. |
| Allocation concealment (selection bias) | Unclear Risk | Not reported |
| Blinding of participants and personnel (performance bias)  All outcomes | High Risk | No assessor-provided intervention; posters, pamphlets, leaflets  Participants not blinded |
| Blinding of outcome assessment (detection bias)  All outcomes | Low Risk | All outcomes self-reported (no assessor rated outcomes). Menstrual knowledge not likely to be influenced by lack of blinding. |
| Incomplete outcome data (attrition bias)  All outcomes | Low Risk | 95% retention rate (84 participants lost of 1689) p. 575 |
| Selective reporting (reporting bias) | Unclear Risk | No trial registration number given. Outcomes only reported in results, methods do not include list of outcomes assessed. |
| Imbalance of outcome measures at baseline | Low Risk | Randomised design. Knowledge of menstrual practice comparable at baseline. |
| Comparability of intervention and control group characteristics at baseline | Low Risk | Randomised design. Characteristics comparable at baseline. |
| Protection against contamination | Low Risk | Cluster randomised design. |
| Other bias | Low Risk | N/A |

***Montgomery 2012***

| **Bias** | **Author’s judgement** | **Support for judgement** |
| --- | --- | --- |
| Random sequence generation (selection bias) | High Risk | “A sample of 120 schoolgirls between the ages of 12 and 18 from four villages in Ghana participated in a non-randomised trial of sanitary pad provision with education” p.e48274  Three periurban sites randomised via coin toss. “After site recruitment, we used a coin flip to assign each site to a different intervention group” (p.e48274).  Rural site non-randomly assigned to pads and education condition. |
| Allocation concealment (selection bias) | High Risk | Non-randomised design |
| Blinding of participants and personnel (performance bias)  All outcomes | High Risk | “Education was delivered in the local language at all three active sites during school hours to groups of 15-25 girls by trained research assistants” |
| Blinding of outcome assessment (detection bias)  All outcomes | Low Risk | “Teachers recorded their attendance daily as usual. Researchers collected attendance records dating from September 2008 to 2009. These dates captured 2 whole terms (1 term=65 days) and one half term. Researchers compared official attendance data with actual student attendance at every site visit (planned and unplanned) and found negligible differences indicating strong reliability of the school attendance data” Montogmery et al., 2013 p.e48274  Self-reported attitudes at higher risk of bias. |
| Incomplete outcome data (attrition bias)  All outcomes | Low Risk | “Analysis was conducted on an intent-to-treat basis…. Missing values were mean-imputed”. “There were 120 girls included in the attendance analysis. Of these 22 were missing complete attendance records, for these data were imputed” p. e48274 |
| Selective reporting (reporting bias) | Low Risk | Trial registration PACTR201202000361337 |
| Imbalance of outcome measures at baseline | Low Risk | "At baseline the pupils in the four sites did not differ significantly (with regard to attendance)" p.e48274  For psychosocial outcomes participants significantly differed at baseline on all subjective measures (shame, lack of self-confidence, insecurity and difficulty concentrating) representing a high risk. |
| Comparability of intervention and control group characteristics at baseline | Low Risk | Intervention and control groups comparable at baseline. Significant differences between rural site and periurban sits in time distance travelled to school and Poverty Index scores. |
| Protection against contamination | Low Risk | Clustered design. |
| Other bias | Low Risk | NA |

***Oster 2010***

| **Bias** | **Author’s judgement** | **Support for judgement** |
| --- | --- | --- |
| Random sequence generation (selection bias) | Low Risk | “a public lottery was carried out and twenty-five girls in each school were assigned to the treatment group” p.4 |
| Allocation concealment (selection bias) | Low Risk | “A public lottery was carried out and twenty-five girls in each school were assigned to the treatment group. Treatment girls remained at the meeting and they (and their mothers) were given a menstrual cup, and instructions on how to use it” p.4 |
| Blinding of participants and personnel (performance bias)  All outcomes | High Risk | “Treatment girls remained at the meeting and they (and their mothers) were given a menstrual cup, and instructions on how to use it” p.4 |
| Blinding of outcome assessment (detection bias)  All outcomes | Low Risk | “Our primary measure of school attendance is official school records, which are collected daily for each student in each school and are available for entire year of the intervention” p.5 |
| Incomplete outcome data (attrition bias)  All outcomes | Low Risk | “One of the mother-daughter pairs randomised to the treatment group decided not to accept the menstrual cup. We analyse the intention to treat effect, and keep this girl in our sample for analysis.” |
| Selective reporting (reporting bias) | Unclear Risk | No trial registration number reported. |
| Imbalance of outcome measures at baseline | Low Risk | Randomised design |
| Comparability of intervention and control group characteristics at baseline | Low Risk | Randomised design |
| Protection against contamination | Low Risk | Girls unlikely to share menstrual cups |
| Other bias | Low Risk | N/A |

***Wilson 2014***

| **Bias** | **Author’s judgement** | **Support for judgement** |
| --- | --- | --- |
| Random sequence generation (selection bias) | High Risk | “it was a partial preference, parallel group, cluster randomised control pilot study. Ten schools in rural Kenya were randomised to either intervention or control).” p.69  “Diagram to represent the restrictions met when randomising schools to intervention arm due to the proximity of the schools. (A) and (b) were in the same area so had to be allocated to the same group. (C) and (d) were in the same area so had to be allocated to the same group. € had to be allocated to the intervention group as another school in the area, not involved with the research, was receiving general teaching by Irise. (F) and (G) were in the same area so had to be allocated to the same group”  Additional online report. Wilson et al., 2012. p.18. |
| Allocation concealment (selection bias) | High Risk | Non-randomised  “Diagram to represent the restrictions met when randomising schools to intervention arm due to the proximity of the schools. (A) and (b) were in the same area so had to be allocated to the same group. (C) and (d) were in the same area so had to be allocated to the same group. € had to be allocated to the intervention group as another school in the area, not involved with the research, was receiving general teaching by Irise. (F) and (G) were in the same area so had to be allocated to the same group”  Additional online report. Wilson et al., 2012. p.18. |
| Blinding of participants and personnel (performance bias)  All outcomes | High Risk | “The intervention group followed questionnaire 1 with a training session on how to make a reusable sanitary pad and were provided with enough equipment to make three pads” |
| Blinding of outcome assessment (detection bias)  All outcomes | High Risk | All outcomes self-reported (no assessor rated outcomes). Self-reported attendance (retrospectively reported for the four weeks prior to assessment) may be influenced by lack of blinding.  “All outcomes were self-reported on questionnaires carried out in class with an Irise facilitator and a local university student acting as a translator” p.70 |
| Incomplete outcome data (attrition bias)  All outcomes | High Risk | “At baseline, 302 pupils were enrolled and 174 were followed-up a month later. Follow-up was possible in nine out of the ten schools” – additional online study report Wilson et al., 2012 p.4  One school from the control condition (N=30) was lost to follow up – additional Wilson et al., 2012 p.22 |
| Selective reporting (reporting bias) | Unclear Risk | No trial registration number reported. Outcomes described in methods reported in results. |
| Imbalance of outcome measures at baseline | Low Risk | Attendance did not differ between intervention and control groups at baseline. |
| Comparability of intervention and control group characteristics at baseline | Low Risk | “Baseline demographics were similar between control and intervention groups” (Wilson et al., 2012, p.21) |
| Protection against contamination | Low Risk | Clustered design |
| Other bias | Low Risk | NA |
