# Supplementary material for: Do Menstrual Hygiene Management Interventions Improve Education and Psychosocial Outcomes for Women and Girls in Low and Middle Income Countries? A Systematic Review
Source: PLoS One. 2016 Feb 10;11(2):e0146985. doi: 10.1371/journal.pone.0146985 (PMC4749306; doi:10.1371/journal.pone.0146985)
Supplement: S3 Table — (DOCX) [file pone.0146985.s003.docx]

**S3 Table**

**Full-text studies excluded with reasons**

| **Author** | **Title** | **Reason for ineligibility** |
| --- | --- | --- |
| Allah 2011 | Impact of health education intervention on knowledge and practice about menstruation among female secondary school students in Zagazig city | No control group |
| Arora 2013 | Impact of health education on knowledge and practices about menstruation among adolescent schoolgirls of rural part of district Ambala, Haryana. | No control group |
| Avachat 2011 | Impact of sex education on knowledge and attitude of adolescent school children of Loni village. | No control group |
| Barua 2010 | Effectiveness of reproductive health education in adolescent, rural schoolgirls of Udupi Taluk, Karnataka | No control group |
| Birdthistle 2011 | What Impact Does the Provision of Separate Toilets for Girls at Schools Have on Their Primary and Secondary School Enrolment, Attendance and Completion?: A Systematic Review of the Evidence | Review, no primary data |
| Chiou 2007 | Effect of systematic menstrual health education on dysmenorrheic female adolescents’ knowledge, attitudes, and self-care behaviours | High-Income Country (Taiwan, China), World Bank 2014 |
| Crofts 2012 | Menstrual hygiene in Ugandan schools: an investigation of low-cost sanitary pads | Qualitative investigation only (no control group) |
| Dongre 2007 | The effect of community-based health education intervention on management of menstrual hygiene among rural Indian adolescent girls | No control group |
| Golbas 2012 | The effect of menstrual health education conducted with peer education method to adolescent girls in 6-8 classes on information and behaviors. [Turkish] | No control group |
| Haque 2014 | The effect of a school-based educational intervention on menstrual health: An intervention study among adolescent girls in Bangladesh | No control group |
| Jasper 2012 | Water and sanitation in schools: a systematic review of the health and educational outcomes | Review, no primary data |
| Jena 2012 | Adolescent girls and reproductive health: An interventional study in a slum of Vijayawada, AP. | No control group |
| Malleshappa 2011 | Knowledge and attitude about reproductive health among rural adolescent girls in Kuppam mandal: An intervention study. | No control group |
| Nemade 2009 | Impact of health education on knowledge and practices about menstruation among adolescent schoolgirls of Kalamboli, Navi-Mumbai. | No control group |
| Rabieipoor 2011 | Empowering of Oromieh University female students in related to their sexual and reproductive health by peer education method. | No control group |
| Rusakaniko 2012 | Trends in reproductive health knowledge following a health education intervention among adolescents in Zimbabwe | No control group |
| Shah 2013 | Improving quality of life with new menstrual hygiene practices among adolescent tribal girls in rural Gujarat, India | No control group |
| Shalini 2011 | Effectiveness of health education programme in raising knowledge, regarding reproductive and sexual health of adolescents among female teachers of senior secondary schools of Amritsar district. | No control group |
| Shirzadi 2012 | The effect of education based on health belief model on promotion of physical puberty health among teen girls in welfare boarding centers in Tehran. [Persian]. | No control group |
| Shrestha 2004 | Effectiveness of reproductive health and aids education among adolescent girls in Udupi Taluk, India | No control group |
| Singh 2013 | Life skill education-an experience of sensitising out of school adolescents about their sexual and reproductive health. | No control group |
| Subhashrao 2010 | Effectiveness of adolescence education programme among adolescent girls and boys: a school based intervention study in Nalgonda District of Andhra Pradesh | No control group |
| Sumpter 2013 | A systematic review of the health and social effects of menstrual hygiene management | Review, no primary data |
| Tartac 2011 | Knowledge of and attitude to menstrual hygiene of students in a vocational school and evaluation of the effectiveness of the training. [Turkish] | No control group |
