# Supplementary material for: Do Menstrual Hygiene Management Interventions Improve Education and Psychosocial Outcomes for Women and Girls in Low and Middle Income Countries? A Systematic Review
Source: PLoS One. 2016 Feb 10;11(2):e0146985. doi: 10.1371/journal.pone.0146985 (PMC4749306; doi:10.1371/journal.pone.0146985)
Supplement: S1 Text — (DOCX) [file pone.0146985.s005.docx]

**S3 Supporting Information. Supplement**

**Other publications arising from this work.**

**Abstract from oral presentation published as:**

Hennegan, J., & Montgomery, P. (2015). Do menstruation management interventions improve education and psychosocial outcomes for women and girls in low and middle income countries? A systematic review. *Journal of Epidemiology & Community Health;69:Suppl 1:* A25-A26 doi:10.1136/jech-2015-206256.38

**Do menstruation management interventions improve education and psychosocial outcomes for women and girls in low and middle income countries? A systematic review.**

Julie M Hennegan*, Paul Montgomery

Centre for Evidence-Based Intervention, University of Oxford, Oxford, United Kingdom

**Background.** Menstrual hygiene management (MHM) is rapidly emerging as an important avenue for interventions seeking to promote gender equality and improve health and wellbeing for women in low and middle income countries. This systematic review aimed to summarise and critically appraise evidence for the effectiveness of menstruation management interventions in improving women and girls’ educational, occupational, and psychosocial wellbeing in low and middle income countries.

**Methods.** Individual and cluster randomised controlled trials were eligible for inclusion, as were non-randomised controlled trials. Education interventions and resource provision interventions (including the provision of sanitary products, improvements to water, sanitation and hygiene, improved privacy, or disposal facilities) were eligible interventions. Education, occupation, and psychosocial outcomes derived from qualitative studies of menstruation management were eligible. Peer-reviewed and grey literature was sought through structured systematic search in 15 databases and complementary hand-searching. Titles and abstracts were screened for eligibility by two reviewers. Risk of bias was assessed by two reviewers using the tool described in the *Cochrane Handbook* and additional items for non-randomised studies suggested by the *Cochrane* *Effective Practice and Organisation of Care* group. Narrative synthesis is provided, and one set of outcomes were able to be synthesised using a random-effects meta-analysis.

**Results.** Searches returned 10,674 unique titles, from these 34 full-text articles were screened. Eight studies described in ten citations met criteria. Six included assessment of education-only interventions, and three assessed the provision of sanitary products (menstrual cups, disposable sanitary pads, and reusable sanitary pads). No trials reported harms. A moderate but non-significant standardised mean difference was found for two studies assessing the impact of sanitary product provision on school attendance: 0.49 (95%CI -0.13, 1.11). Included studies were heterogeneous with considerable risk of bias. Trials of education interventions reported positive impacts on menstrual knowledge and practices, however, many studies failed to assess other relevant outcomes.

**Conclusions.** There is insufficient evidence to fully establish the effectiveness of menstruation management interventions, although current results are promising. Eight trials have been conducted, but a high risk of bias was found and clinical heterogeneity precluded synthesis of most results. Whilst trials provided some indication of positive results, further research is needed to establish the role of menstruation hygiene management in education performance, employment and other psychosocial outcomes. This review provides a concise summary of present trials and highlights important improvements for future work.
